# Small Target Detection in Refractive Panorama Surveillance Based on Improved YOLOv8

**DOI:** 10.3390/s24030819

**Published:** 2024-01-26

**Authors:** Xinli Zheng, Jianxin Zou, Shuai Du, Ping Zhong

**Affiliations:** College of Science, Donghua University, Shanghai 201620, China; 2212313@mail.dhu.edu.cn (X.Z.); zsdu996@163.com (J.Z.); 1219782@mail.dhu.edu.cn (S.D.)

**Keywords:** YOLOv8, small object detection, reflective panorama, panoramic detection

## Abstract

Panoramic imaging is increasingly critical in UAVs and high-altitude surveillance applications. In addressing the challenges of detecting small targets within wide-area, high-resolution panoramic images, particularly issues concerning accuracy and real-time performance, we have proposed an improved lightweight network model based on YOLOv8. This model maintains the original detection speed, while enhancing precision, and reducing the model size and parameter count by 10.6% and 11.69%, respectively. It achieves a 2.9% increase in the overall mAP@0.5 and a 20% improvement in small target detection accuracy. Furthermore, to address the scarcity of reflective panoramic image training samples, we have introduced a panorama copy–paste data augmentation technique, significantly boosting the detection of small targets, with a 0.6% increase in the overall mAP@0.5 and a 21.3% rise in small target detection accuracy. By implementing an unfolding, cutting, and stitching process for panoramic images, we further enhanced the detection accuracy, evidenced by a 4.2% increase in the mAP@0.5 and a 12.3% decrease in the box loss value, validating the efficacy of our approach for detecting small targets in complex panoramic scenarios.

## 1. Introduction

The field of view in conventional optical system cameras is constrained. As a result, they can only record a limited set of data. However, when covering a vast area for observation, people’s observational needs frequently exceed these restrictions. With their 360-degree field of vision, omnidirectional imaging or panoramic cameras [1] offer an efficient option. A far more comprehensive range can be seen by mounting panoramic cameras from great heights for overhead views, or using drones for aerial patrols. This quality of panoramic cameras gives them a broad range of potential applications, such as simultaneous localization and mapping (SLAM), missile tracking systems, security surveillance for large outdoor warehouses, visual tracking of drones, visual surveillance of border defense equipment, and other fields [2].

Panoramic cameras achieve this vast field of view through designed optical components like fisheye, annular, reflecting lenses, or multi-camera image stitching. Since the first reflective panorama system’s introduction in 1997 [3], panoramic imagery has seen significant advancements, especially in producing high-quality, real-time images encompassing a full 360-degree horizontal view. Despite these advancements, detecting small targets in panoramic images poses unique challenges. Compared to regular photographs, panoramic images have lower resolution per object due to their wide field of view, increasing the complexity of the background and making small target detection more difficult. Addressing these challenges is crucial, especially as limited public datasets are available for catadioptric panoramic images. Our study focuses on enhancing the precision of small target detection in panoramic images and meeting real-time detection requirements.

Current methods for panoramic target identification predominantly focus on prominent targets, leaving a significant gap in the research that explicitly targets the panoramic detection of small objects. Our study aims to address this need and enhance the precision of small target detection in panoramic images, while meeting real-time detection requirements. We have made several key advancements in this area. The main contributions of this study are as follows:

(1) We propose a novel data augmentation method for panoramic images involving copy–paste data. This technique significantly increases the number of training set samples for small targets, ultimately enhancing the model’s ability to detect small targets within panoramic photographs.
(2) We advanced the original YOLOv8s [4,5] network by integrating a specialized tiny target detection layer and replacing the C2f layer in the backbone network with a C2f_EAM layer that incorporates the EAM attention mechanism. We specifically applied these enhancements to the YOLOv8 network.
(3) We upgraded the eighth layer in the network to the FasterNext configuration. This development improves the model’s accuracy and convergence rate and reduces the computational load required.
(4) Our study incorporated a preprocessing step for panoramic images involving planar unfolding, truncation, and stitching. This technique significantly improved small target detection in panoramic images.

## 2. Related Work

Over the past few decades, significant research has focused on small target detection, a field challenged by the limited size, texture, and shape information these targets provide, often ranging from just a few to dozens of pixels. This has led researchers to continually refine detection algorithms for small targets, aiming to strike a balance between detection speed and model accuracy.

In the early stages of target detection, methods often relied on manually designed features combined with detection classifiers. However, since 2012, the rapid development of convolutional neural networks (CNN) has spurred a shift towards deep learning-based object detection algorithms. The two-stage target detection methodology, starting with the region-based convolutional neural network (R-CNN) in 2014 and evolving to Fast R-CNN and SPPnet [6,7], generates candidate regions and classifies them. Though offering high detection accuracy, these methods tend to be slower. In contrast, single-stage target detection algorithms like YOLO (You Only Look Once), SSD (Single-Shot Detector) [8,9], and DETR (Detection Transformer) [10], prioritize speed but often at the expense of accuracy.

Researchers have proposed various methodologies to enhance the detection of small targets. For instance, they have introduced deformable convolution [11] to improve the process of sampling the object size and shape, which addresses the limitations of fixed convolution in extracting spatial information. The feature pyramid network (FPN) [12] development aimed to preserve richer scale features, thus bolstering small target detection. Gong Y and colleagues devised a fusion factor to balance the information transfer between deep and shallow layers of the image pyramid, enhancing the learning efficiency for small targets [13]. Additionally, they proposed the Normalized Wasserstein Distance (NWD) as a scale-insensitive method for measuring similarity [14]. They developed feature super-resolution (FSR) [15] as an alternative to traditional image super-resolution approaches.

Several researchers have addressed the unique challenges in the context of panoramic imaging. Pengyu Zhao [16] and the team proposed the reprojected R-CNN for rapid and precise detection in 360° panoramic images. Junjie Wu [17] and his team developed a method using the Sample Adaptive View Transformer (SAVT) module, enhancing notable target detection in 360° panoramic images. Pengfei Jia [18] and others improved the YOLOv4 algorithm for panoramic video target detection. At the same time, Olfa Haggui [19] and colleagues developed a color histogram comparison model based on the Bhattacharyya distance for fisheye image tracking. Cai Chengtao’s team improved the YOLO algorithm for real-time multi-object monitoring in catadioptric panoramic images. Hongzhen Xu [20] and others used the Lucas–Kanade optical flow algorithm for moving obstacle detection in panoramic images. Ma Ziling and colleagues utilized a differential block elimination model for motion target detection. Yuhang He [21] introduced a multi-modal target tracking framework that leverages 2D panoramic images and 3D point clouds.

## 3. Methodology

**Figure 1:**
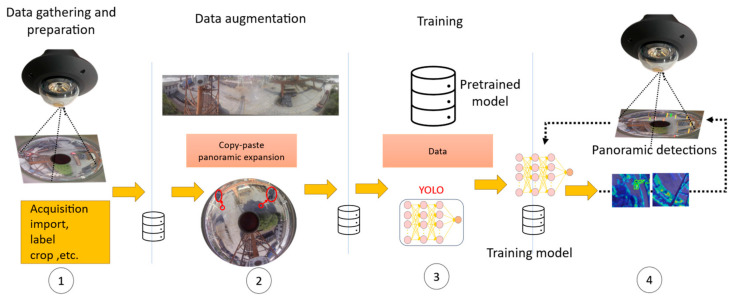
The general training and generalization process for panoramic image small target detection involves four steps: ① the acquired images are annotated using the LabelImg tool and then cropped to conform to the YOLOv8 model’s input format; ② data augmentation is utilized to address sample imbalance and to expand the dataset after panoramic expansion; ③ these processed images are used to train the YOLOv8 detector; ④ the trained model is deployed to detect small targets in panoramic images, producing the final visualization of the detection results.

Figure 1 depicts the workflow utilized in this study for detecting small targets within custom-made panoramic datasets. The diagram conveys the end-to-end process, from the original panoramic image acquisition to the final detection output. The annotation of raw dataset images is conducted using the LabelImg tool, preparing them for compatibility with the YOLOv8 model’s requirements. Following this, data augmentation strategies are implemented to correct sample imbalances and to enrich the dataset post-panoramic expansion. The YOLOv8 detector is then trained on these annotated and processed images. Finally, the fully trained model is applied to previously unseen images for detecting small targets, culminating in a comprehensive visualization of the detected targets.

### 3.1. YOLOv8s Network Architecture

The YOLOv8 network [5,22], which debuted in 2023 and is the most recent iteration of the YOLO architecture series [4], does away with the bounding box operation that YOLOv5 [23,24,25] utilized. Its primary input is 640 × 640, and depending on the scaling factor, the network offers different size models with N, S, M, L, and X dimensions to accommodate the requirements of a wide variety of use cases. When the YOLOv8s model was put through its paces using the official COCO dataset, the mAP@50-90 accuracy value reached 44.9, and the CPU inference speed was measured at 128.4 milliseconds.

**Figure 2:**
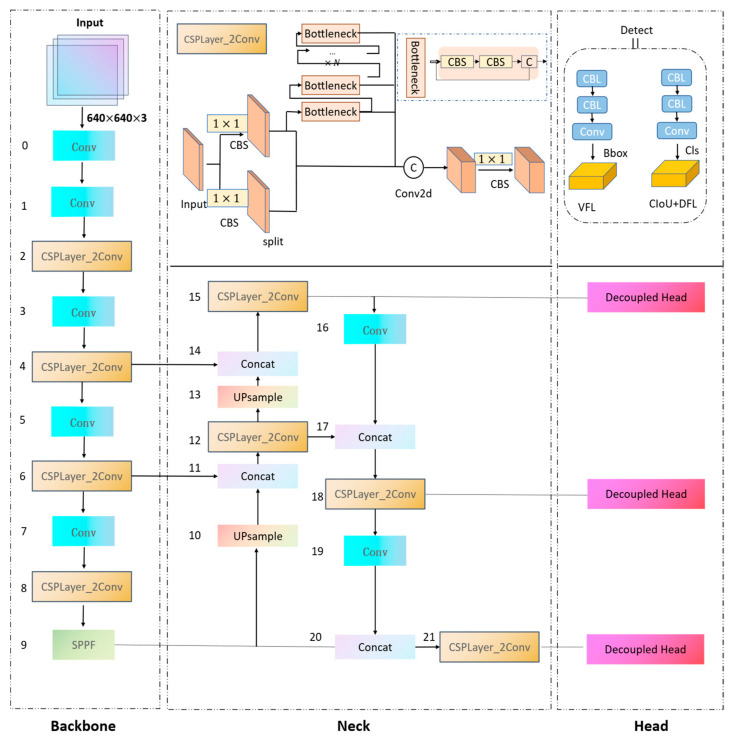
The network structure of YOLOv8s and the CSPLayer_2Conv (C2f) structure in the YOLOv8 network, and the detection header structure in YOLOv8.

The high detection accuracy of YOLOv8 stands as one of its most notable features, with YOLOv8s demonstrating a 7.5% improvement in model accuracy compared to YOLOv5s. While the inference speed of the YOLOv8s model is marginally lower than that of YOLOv5s, it significantly excels in regard to accuracy, especially for non-small objects. However, it was found that the standard YOLOv8 model is less effective in accurately detecting small targets. This limitation led us to focus our research on enhancing small target detection. The structure of the YOLOv8s network, which our research is based on, is depicted in Figure 2. Our enhancements, while not visually represented in Figure 2, aim to optimize the YOLOv8s architecture to improve the precision, accuracy, and recall of small target detection in wide-area panoramas, with minimal impact on the detection speed. These advancements have been instrumental in refining the network’s capability for small target detection in complex panoramic scenes.

The YOLOv8s architecture comprises three main components: the backbone network, neck network, and detection network. For the backbone network, YOLOv8s specifically utilizes DenseNet-53 for its backbone network, chosen for its robust feature extraction capabilities. This architecture incorporates the C2f module instead of the standard C3 module, as depicted in Figure 2. The C2f module, inspired by CSPNet’s bypass extraction concept, integrates with the residual structure and consists of three convolution modules (Conv+BN+SiLU) and n bottlenecks. Such configuration allows YOLOv8s to ensure a lighter network, while facilitating richer gradient flow information.

The YOLOv8 model employs a bounding box-free operation. As shown in Figure 2, the structure of the head section visibly draws inspiration from the design concepts of YOLOX and YOLOv6 [26,27] and implements a decoupled-head structure. This structure uses two convolution layers to perform classification and regression operations. Specifically, the upper branch is responsible for the convolution regression of the bounding box, while the lower limb handles the convolution classification of the categories. Worth noting is that, compared to YOLOv5, the YOLOv8 model eliminates the obj_loss branch, thereby negating the need for a grid confidence calculation. Additionally, the number of channels in the regression head is set to be four times the maximum regression value (reg_max).

The YOLOv8 model has adopted a novel strategy for sample matching. It discards the traditional IOU matching or unilateral ratio allocation method, instead using the task-aligned assigner for positive and negative sample matching, and introducing Distribution Focal Loss (*DFL*) [28] (as shown in Equation (1)).

(1) $$DFL\left(S_{i}S_{i+1}\right)=-(\left(y_{i+1}-y\right)\log\left(S_{i}\right)+(y-y_{i})log(S_{i+1}))$$

Equation (1) modifies the conventional focal loss by considering the distribution of the predicted value around the target class y. Here, $S_{i}$ and $S_{i+1}$ represent the scores of the adjacent classes of the actual class y. The term $y_{i+1}-y\log\left(S_{i}\right)$ weighs the log likelihood of the lower adjacent class $S_{j}$ by the difference between the upper adjacent class $y_{i+1}$ and the actual value y, while $\left(y-y_{i})\log(S_{i+1}\right)$ similarly weighs the likelihood of the higher adjacent class $S_{j+1}$. Through this, *DFL* allows the network to adaptively focus on the prediction errors closer to the actual value, thereby improving the accuracy of bounding box localization.

For the classification loss function, YOLOv8 incorporates Versatile Focal Loss (VFL), as formulated in Equation (2):

(2) $$VFL\left(p,q\right)=\left\{\begin{array}{cc} -q\left(q\log\left(p\right)+\left(1-q\right)\log\left(1-p\right)\right) & q>0\\ -\alpha p^{\gamma }\log\left(1-p\right) & q=0 \end{array}\right.$$

*VFL* applies an asymmetric weighting operation to adjust the loss based on the label *q*. When *q* > 0, indicative of a positive sample, the loss is calculated using a weighted combination of the predicted probability p and the adaptive  IOU  weighting q, emphasizing the agreement between the predicted box and the ground truth; for negative samples where q=0, the term $-\alpha p^{\gamma }\log\left(1-p\right)$ reduces the loss for well-classified negative samples, preventing them from dominating the gradient. This selective focus on positive samples and their alignment with the ground truth aids in refining the classification accuracy.

### 3.2. Addition of Small Object Detection Layer

To enhance the detection of small objects in panoramic images, we can understand from the network architecture of YOLOv8s that the input image size is 640 × 640, and the output feature size is 80 × 80, 40 × 40, 20 × 20. When the size of the target object is 30 × 30, the corresponding size of the feature map is 3.75, 1.875, 0.9375. If it is less than 1, the feature does not exist. Therefore, the smaller the target size, the weaker the detection capability.

**Figure 3:**
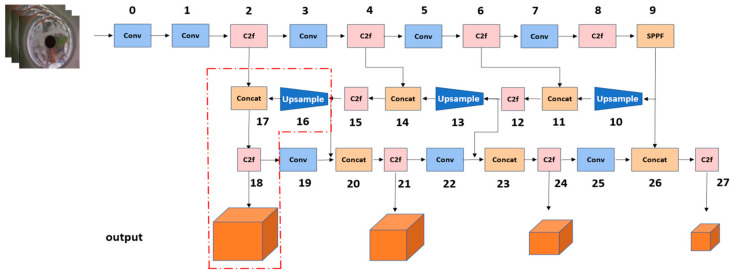
Schematic structure of the small object detection layer added to YOLOv8s.

Processing panoramic images presents a unique challenge: the expansive field of view necessitates the detection of smaller targets. We have enhanced our network to integrate small target detection methodologies more deeply to address this. Figure 3 illustrates that a new branch has been added after the second layer, C2f. The area highlighted in red signifies our optimization, introducing a 160 × 160 feature map to the output. This advancement facilitates more accurate detection of smaller targets by preserving extensive feature information during training. As the network deepens, however, there is a potential loss of valuable small target information, and the model’s size may increase, slowing the detection speed. To counter this, we incorporated the multiscale attention (EMA) mechanism and the FasterNext module in subsequent steps. This allows the network to concentrate more effectively on small target information, enhancing the overall detection accuracy and efficiency, while reducing the operational speed.

### 3.3. Integration into the FasterNext Network

To enhance model training efficiency and minimize the number of floating-point operations (FLOPs), Jay Chen et al. [29] introduced the faster neural network (FNN), a novel network that significantly bolsters the operational speed of the network without compromising the accuracy of visual tasks. Inspired by their work, we incorporated FNN into YOLOv8. We further improved the faster neural network by replacing the C2f network structure at layer 8 in the YOLOv8s model with an enhanced FasterNext module. The design of the FasterNext neural network proceeds as follows.

Due to frequent memory access, traditional convolution often needs to be more efficient in terms of computational speed and floating-point operations (FLOPs). Despite being widely adopted as a fundamental building block for neural networks, depthwise convolution (DWConv), a variant of Conv, also presents significant challenges. We have utilized the FasterNext network to address these issues, which employs parametric convolution (PConv) [30]. This approach allows more efficient and effective use of the device’s computational power to extract spatial features.

**Figure 4:**
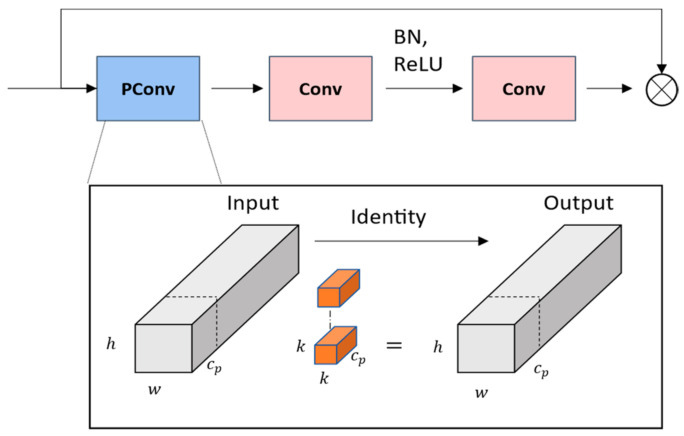
FasterNext network structure.

As shown in Figure 4, regular Conv is applied for spatial feature extraction only on the part of the input channel, without affecting the rest of the channels; for sequential or regular memory access, we consider the first or the last consecutive c_p channel as a representation of the whole feature map used for the computation; the input and the output feature maps have the same number of channels: $h\times w\times k^{2}\times {c_{p}}^{2}$ and the ratio of Conv to $r_{c}=0.25$, and the ratio of $r_{FLOPs}=0.0625$. The PConv memory is only 1/4 of the Conv. The FasterNext block consists of a single PConv layer, which, together with the two Convs, forms an inverted residual block, where the intermediate layer has an extended number of channels, and shortcut connections are placed in order to reuse the input features. Conv layers are placed between the normalization and activation layers to maintain feature diversity and achieve lower latency.

### 3.4. Integration into the Multiscale Attention (EMA) Module

The channel or spatial attention mechanism can be used to achieve good feature representation, but the channel dimensionality reduction it brings will have some side effects when extracting the depth visual representation. Daliang Ouyang et al. [31] proposed a new efficient multiscale attention module that reduces the operations, while preserving the information of each channel. We add the EMA mechanism to the C2f module in the backbone network, and C2f_EMA replaces the original backbone C2f module.

**Figure 5:**
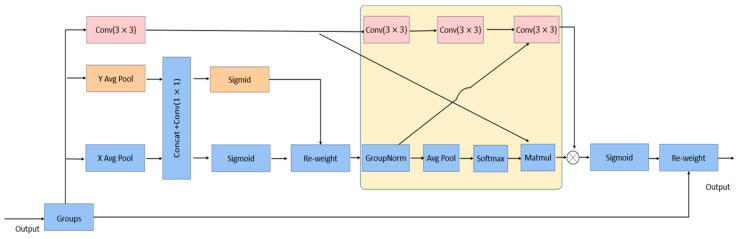
EMA module structure.

As detailed in Figure 5, the EMA module adopts a parallel structure, which can be divided into three branches, two 1 × 1 and 3 × 3 branches, to extract the attention weight descriptors of the grouped feature maps, where the 1 × 1 branch references the revisit coordinate (CA) [32] module. The CA input tensor is decomposed into two parallel one-dimensional feature encoding vectors, along the horizontal and vertical dimensional directions. Complete set average pooling, using spatial location information, is used to model cross-channel correlations, no dimensionality reduction convolution in 1 × 1; 2D binary distribution on linear convolution is fitted with a Sigmoid function after decomposing the output of the 1 × 1 convolution into two vectors. Only a 3 × 3 kernel is stacked in the 3 × 3 branch to capture multiscale feature representations to expand the feature space.

Global spatial information is then encoded in the output of the 1 × 1 branch using group normalization and 2D global pooling operations. The natural nonlinear function Softmax, using 2D Gaussian mapping, is used at the back of the 2D global pooled output. The processed output is then multiplied with the matrix dot product operation after convolution in the 3 × 3 branch to obtain the first spatial attention map. The global spatial information from the 3 × 3 branches is encoded using 2D global average pooling, and the output of the smallest branch will be converted into the corresponding dimensional shape directly before the joint activation mechanism of the channel features, in order to derive the second spatial attention map that preserves the entire precise spatial location information. Finally, the output feature maps within each group are computed as an aggregation of the two generated spatial attention weight values, followed by a Sigmoid function. It captures pixel-level pairwise relationships and highlights the global context of all the pixels.

**Figure 6:**
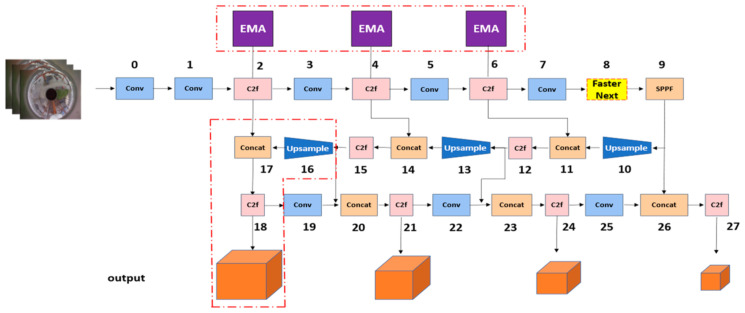
Improved network based on YOLOv8.

Figure 6 illustrates the schematic structure of the added small object detection layer in the YOLOv8s architecture. In this structure, we have optimized explicitly for detecting small objects within panoramic images. Traditional feature map sizes may fail to capture smaller target objects, so we introduced a higher-resolution feature map output to preserve more detailed feature information. As shown in Figure 6, a new branch has been added following the second layer C2f, with the area highlighted in red indicating our optimization strategy, introducing a 160 × 160 feature map to the output. This approach allows for more accurate detection of small targets during training. As the network delves into deeper layers, there is a potential risk of losing crucial information about small targets, which could also lead to an increase in the model size and possibly a decrease in the detection speed. We have strategically integrated the multiscale attention (EMA) mechanism to address these challenges at the 2nd, 4th, and 6th C2F layers. Additionally, we replaced the eighth layer C2f with the FasterNext module. These modifications enable the network to focus more effectively on small target information, thereby improving detection accuracy and optimizing computational efficiency.

## 4. Experiments

### 4.1. Panoramic Image Acquisition

**Figure 7:**
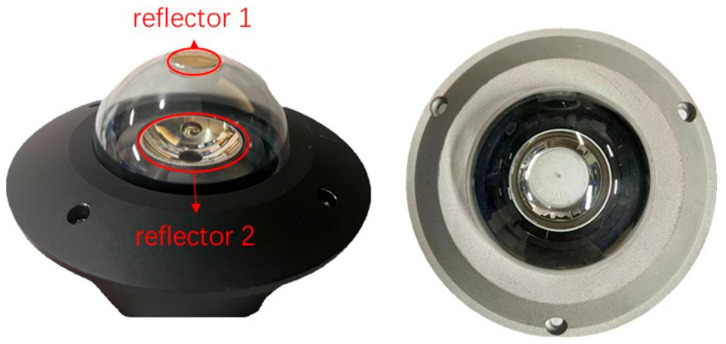
Physical image of the hyperbolic refractive panoramic camera, where reflector 1 and reflector 2 are shown on the upper and lower surfaces.

There is a growing need for larger, more complex, high resolution, and accurately annotated datasets for object detection in reflective panoramic images [1]. To address this need, this study concentrates on small targets within retroreflective panoramic images and has established a dedicated dataset repository specifically for these types of images. The dataset was gathered at a large steel warehouse located in the Tiexi district of Shanghai. Figure 7 displays the physical image of the hyperbolic retroreflective panoramic camera used for data collection.

The panoramic camera was mounted on a 20 m tower crane, covering an area of at least 7000 m^2^. Two scenes were captured with five different pixel panoramic images. One set of images had a resolution of 2596 × 1920 pixels, with small targets occupying only 3427 pixels, accounting for only 0.018% of the entire image. The other set of images had a resolution of 1920 × 1080 pixels, with people occupying only 23 × 11 pixels in the image. The retroreflective panoramic image collection process intermittently collected images for one month, from morning to night, capturing one image every half an hour, including in sunny, cloudy, and rainy weather. A total of 4382 panoramic images were obtained with 80 pixels, with people occupying only 23 × 11 pixels in the image. A total of 4382 panoramic images were obtained using the BMP image format.

**Figure 8:**
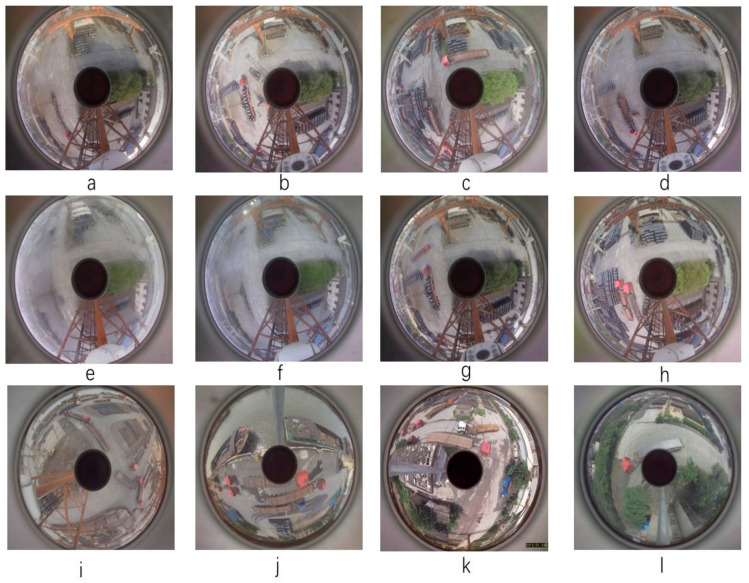
Images captured by a refractive panoramic camera of different scenes under varying light and weather conditions. (**a**) Clear day with moderate lighting, (**b**) Sunny day with strong shadows, (**c**) Overcast conditions, (**d**) Overcast evening, (**e**) Morning with fog and dew, (**f**) Foggy condition with artificial lighting, (**g**) Overcast with diffuse illumination, (**h**) Bright sunny day, (**i**) Overcast day with heavy traffic, (**j**) Sunny day with reflective surfaces, (**k**) Rainy conditions with wet surfaces, (**l**) Green outdoor setting with natural light.

Figure 8a–h shows images captured at different periods of the same scene, while Figure 8i–l shows panoramic images captured of different scenes. Observing the data, we found three significant problems in retroreflective panoramas: 1. various forms of distortion; 2. discontinuous edge effects; 3. changes in object scale. Therefore, we must improve the object detection algorithm to improve the model’s tolerance for distortion, edge effects, and object scale features.

**Figure 9:**
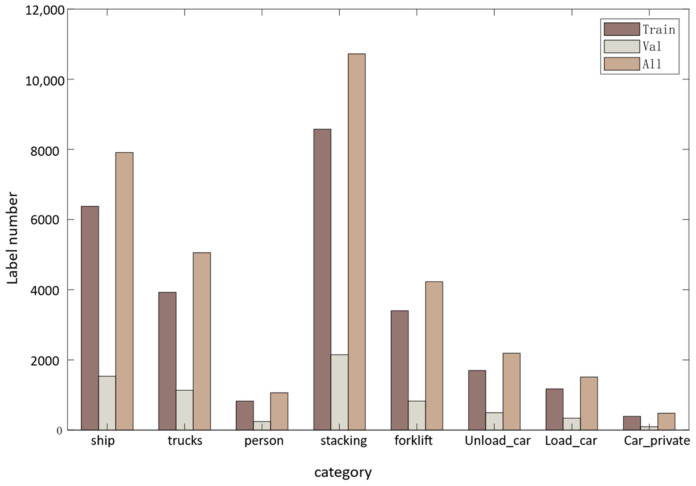
Number of tags per category in the panorama dataset.

In addition to people (small targets), we labeled seven other objects with different scales during the panoramic image labeling process, namely trucks, ships, steel piles, cars, loaded and unloaded truck compartments, and forklifts. Due to the uneven distribution of the collected dataset, there is more data on steel piles and ships, with 10,721 and 7906 objects labeled, respectively. In contrast, the datasets on cars and people are relatively small, with 1062 and 477 objects labeled, respectively. The edges of the panoramic images have significant deformation, and the ships in the dataset are located on the edges of the images and are easily occluded. The size of the people also changes according to their distance from the camera, leading to changes in scale. We randomly selected 3560 images from the 4382 panoramic images as the training set and 866 as the validation set. The distribution of the data labels in the training and validation sets is shown in Figure 9.

### 4.2. Image Preprocessing

Because deep learning requires a large amount of datasets to obtain the feature information of images, the categories of the collected panoramic datasets are unevenly distributed, and there are insufficient small target samples. Therefore, we augmented the existing datasets. Due to the limited data samples featuring humans, we used an improved image copy–paste method [33] to expand the tiny target (human) sample labels to obtain more comprehensive feature information, thereby enhancing detection accuracy.

**Figure 10:**
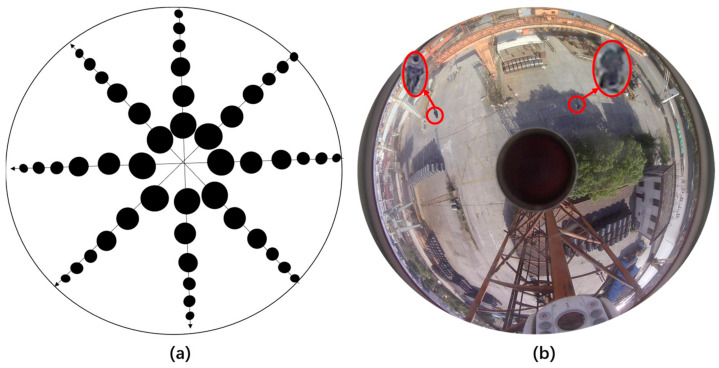
Schematic of the panoramic copy–paste data enhancement approach. (**a**) Schematic of copy–paste method in panoramic view; (**b**) the result of the copy–paste model in the panorama is realistic.

We first extract the small target model (human) through the SAM (Grounded-Segment-Anything) [34] model, randomly rotate it from 0–45°, and scale it according to the distance from the center point of the panoramic image, randomly generating 716 small targets, and randomly integrating them into different panoramic image training sets. In this way, the training set of small targets is expanded. If too many are copied, this random copying may block the original data, and the overall detection accuracy will decrease. Moreover, the integrated samples are only present in the training set, not the test set. Figure 10a is a schematic diagram of the copy–paste method for panoramic images. The person circled in Figure 10b is integrated into the panoramic image through the copy–paste method, expanding the sample labels of the small targets.

To further expand the dataset and enhance the robustness of our model, we leveraged the data augmentation technology built into YOLO [4]. Given the spherical nature of panoramic images, we employed a range of image processing techniques to augment the data. These techniques include image rotation, mirroring, HSV space channel enhancement, scaling, multiscale transformation, blending, cropping, and segment copy–paste. These modifications enable the model to better adapt to diverse scenarios.

**Figure 11:**
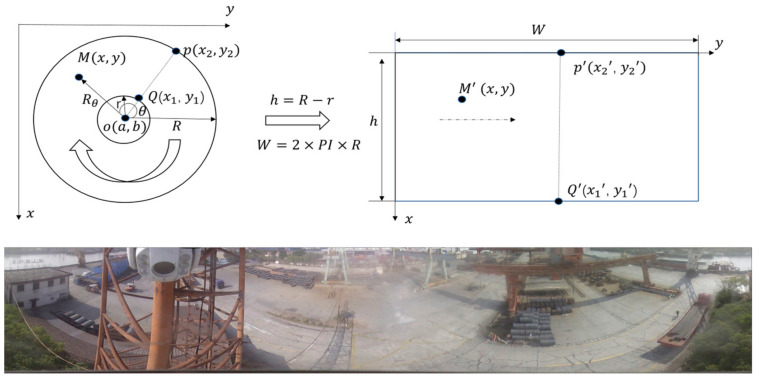
Detailed schematic of panoramic image unfolding.

For a single viewpoint, complete reflection panoramic image, each pixel corresponds to a light ray that intersects the viewpoint. By employing the parameters of the panoramic imaging system, we project these pixels onto a cylindrical surface or a plane, unfolding the image. This unfolded representation articulates 360° of the horizontal spatial information, which significantly enhances the efficacy of the subsequent processes, such as small target detection and other computational tasks. Our research predominantly utilizes the plane unfolding method for data preprocessing of the complete reflection panoramic image. Figure 11 shows a two-dimensional rectangular, cylindrical panorama from the original image. The center of this panorama is denoted, with R representing the radius. There exists a precise mathematical relationship between the coordinates of a point, *M*(*x*,*y*), on the panoramic image and its corresponding point, *M*(*x*′,*y*′), on the unfolded image.

(3) $$R_{\theta }=\sqrt{{\left(x-a\right)}^{2}+{\left(y-b\right)}^{2}}$$

(4) $$\theta =arcos\left(\frac{x-a}{R_{\theta }}\right)$$

The radial distance from the center to the point *M*(*x*,*y*) is determined by Equation (3). The radian, which is the projection angle onto the unfolded plane, is calculated by Equation (4).

The coordinates of the expanded image point *M*(*x*′,*y*′) are determined by the transformation of Equations (5) and (6):

(5) $$x^{\prime }=\theta \times R=arcos\left(\frac{x-a}{R_{\theta }}\right)\times R$$

(6) $$y^{\prime }=R-R_{\theta }=R-\sqrt{{\left(x-a\right)}^{2}+{\left(y-b\right)}^{2}}$$

Equation (5) represents the transformation of the *x* coordinate from the original panoramic image to the unfolded image. The angle formed by the radial line from the center to M(x,y) in the panoramic image concerning the horizontal axis. *R* is the radius of the cylindrical panorama, which is a constant for a given panoramic system. Where *x*′ is the new *x* coordinate on the unfolded image and is the radial distance from the center to the point M(x,y), as calculated in Equation (6). And *y*′ is the new *y* coordinate on the unfolded image.

Most panoramic video detections rely on unfolded panoramic images. Since the aspect ratio of the image after unfolding is 2πR/(R − r) ≈ 23.14970/(970 − 228) = 8.2, if the training is carried out directly, the image will be compressed into 640 × 640, resulting in a severe imbalance in the aspect ratio and serious image distortion. We cut the image into two parts and stitched them together after the panoramic view was unfolded to minimize distortion due to image compression. To streamline the creation of an unfolded panoramic dataset, we automated labeling objects in the unfolded images. This was achieved by utilizing the mapping relationship between the unfolded and the original panoramic images. This approach enabled us to efficiently produce processed panoramic datasets with minimized distortion and enhanced accuracy.

### 4.3. Training Platforms

This experiment relies on an ASUS TUFz690 model computer (Intel(R)Core(TM) I7-12700k CPU; 3187 Mhz, 32 GB; NVIDIA Geforce RTX3080 GPU, 12 GB video memory), using the Windows 10 system, Python 3.9, CUDA 11.7, the deep learning framework PyTorch 2.0.0, and OpenCV 4.7.0 to implement the folded reflection panoramic small target detection model training and testing in real-time. In this study, an end-to-end approach is used to train the improved YOLOv8s network using stochastic gradient descent (SGD); the batch size of the model training is set to 8, the learning rate lr0/f = 0.01, and the SGD momentum factor is set to 0.937. The data enhancement coefficients for the image left–right flip (probabilistic), image splicing, image obfuscation, and segmented copy–paste (probabilistic) are 0.5, 1.0, 0.1, 0.1, and 0.1; the training batch is set to 300 rounds, and the resulting recognition model weights file is saved after the training is completed, where early abort is set.

### 4.4. Key Evaluation Indicators

In assessing the performance of the enhanced YOLOv8s network, we adopted standard metrics that are essential for evaluating object detection models. As defined by Equation (7), precision is the ratio of correctly identified positive detections to the total number of detections made. This metric is crucial in understanding how often our model’s predictions are accurate when it claims to have detected a panoramic target.

(7) $$p=\frac{TP\left(True\;Positive\right)}{TP(True\;Positive)+FP(Fales\;Positive)}\times 100\%=\frac{TP}{ALL\;Detection}$$

Here, a true positive (*TP*) occurs when a predicted panoramic tiny target corresponds accurately to a labeled panoramic target. In contrast, a false positive (*FP*) occurs when the model incorrectly identifies the background as a panoramic target.

Recall, given by Equation (8), measures the proportion of actual panoramic targets the model correctly detects out of all the ground truths. This metric provides insight into the model’s ability to find all the relevant instances in the dataset.

(8) $$Recall=\frac{TP\left(True\;Positive\right)}{TP\left(True\;Positive\right)+FN\left(Fales\;Nagative\right)}\times 100\%=\frac{TP}{ALL\;Ground\;Truths}$$

Moreover, to encapsulate the overall prediction accuracy across various intersection over union (IOU) thresholds, we calculate the mean average precision (mAP) as formulated by Equation (9). This comprehensive metric considers the precision–recall balance across different detection thresholds, providing a holistic view of the model’s performance using our panoramic dataset.

(9) $$mAP=\frac{1}{\left|Q_{R}\right|}\sum_{q=i}^{N}P(q)∆R(q)$$

In Equation (9), *q* indicates the IOU threshold, *N* represents the total number of IOU thresholds considered, and $Q_{R}$ is the set of detected targets across all the thresholds.

## 5. Results and Discussion

### 5.1. Effects of Improved Network Modeling

To evaluate the effectiveness of the small target detection layer, the FasterNext module, and the EMA module in detecting small targets in panoramic images, we initially test the panoramic images enhanced through data augmentation, but not those unfolded for stitching. Ablation experiments were performed using the YOLOv8 model, with an input image size of 640 × 640 pixels, targeting eight different types of objects. Table 1 shows the detection results for all these target types, demonstrating the improved model’s enhanced performance in detecting small targets across various panoramic scenes.

According to Table 1, we have an mAP@0.5 of 87.2% for YOLOv8s using the same set of folded-reflection panorama datasets with the same conditions, and the grid optimization of the number of parameters and the model size for the mAP@0.5 value and the network structure results in a significant improvement.

By replacing the C2f module in layer 8 of the original backbone network with the FasterNext module, PConv replaces Conv due to the addition of FasterNext, reducing the floating-point operations by 1.7GFLOPs, the number of parameters by 1.03 M, and the model size is reduced by 2.1 MB.

With the addition of the small target detection header to the YOLOv8s network, the overall mAP@0.5 value increases by 2.3%, and the mAP@0.5 of the tiny target (human) increases by 17.7% to 53.9%, which effectively captures the features of the tiny target and enhances the detection of the small target. However, due to the addition of the tiny target detection layer, the number of floating-point operations is increased by 0.8GFLOPs compared to the YOLOv8s model, with the consequent increase in the number of floating-point operations. Adding a small target detection layer to the YOLOv8_fasterNext network increases the overall accuracy of the mAP@0.5 by 2.9% and the detection accuracy of small targets of the mAP@0.5 by 24.7%.

We incorporate the EMA mechanism into layers 2, 4, and 6 of C2f in the backbone network of YOLOv8. We find that the YOLOv8 network incorporating the multiscale attention EMA mechanism helps to capture cross-dimensional interactions and establish dimensional dependencies efficiently, and that it highlights the global context of all the pixels, and improves the mAP@0.5 of the target detection in panoramic images by 0.4%, and the mAP@0.5 of small target detection goes up by 24.7%. YOLOv8s_small increases the overall mAP@0.5 by 0.5% and the detection rate of small targets (people) by 4.0%, after incorporating the EMA module.

When the YOLOv8s_small_fasterNext_EMA model is compared with the YOLOv8s model, the overall accuracy is improved by 2.9%, in which the mAP@0.5 of the small targets is improved by 20.0%, the model parameter is 1.3 M less, and the size of the model is reduced by 2.4 MB.

We used Grad-CAM as a visualization tool for our network, and the region of most interest to the network is the darker color in the region map. The improved YOLOv8s_small_fasterNext_EMA network outperforms the original YOLOv8s model under the same lighting and environmental conditions.

**Figure 12:**
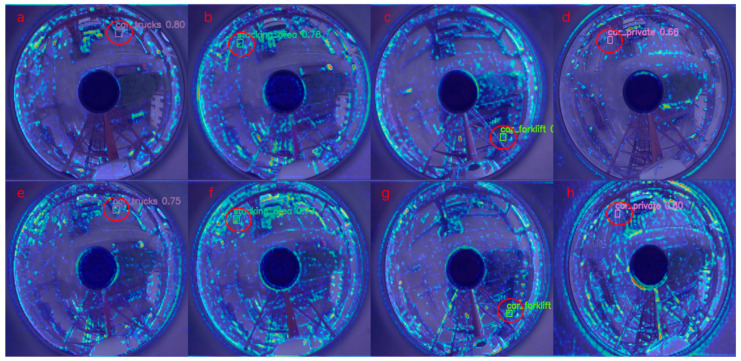
Heat maps: (**a**–**d**) YOLOv8s; (**e**–**h**) YOLOv8s_small_fasterNext_EMA.

Figure 12a–d illustrates the heat maps generated by the original YOLOv8s model, and Figure 12e–h depicts the heat maps from the YOLOv8s_small_fasterNext_EMA model. This side-by-side arrangement of the heat maps allows for a direct comparison between the models regarding the focal intensity on the detected objects. Notably, Figure 12e–h reveals a deeper color intensity within the red-circled regions, signifying a heightened focus of the YOLOv8s_small_fasterNext_EMA model on areas containing small objects. This suggests an enhancement in the model’s capacity to detect and home in on smaller objects within panoramic images. When comparing corresponding Figure 12a with Figure 12e,b with Figure 12f,c with Figure 12g,d with Figure 12h, the increased attention to relevant target areas by the improved model becomes evident, highlighting its superior detection performance. The discernible difference in color depth between the original and the enhanced model’s heat maps accentuates the progress made in pinpointing and delineating smaller objects amidst complex panoramic scenes.

### 5.2. Comparison with Other Algorithms in the YOLO Series using the Panoramic Dataset

We compared the currently available models and our improved models using the panoramic dataset without data enhancement and any processing, and we can draw the following conclusions from Table 2.

Compared with YOLOv8s and YOLOv5s [24,35,36], the overall accuracy mAP@0.5 of YOLOv8s is 1.4% smaller than that of YOLOv5s, the detection accuracy capability related to the small target (person) by YOLOv8s is 16.7% smaller than that of YOLOv5s, and the detection accuracy related to the rest of the categories is better than that of YOLOv5. We improve the model based on the basic model of YOLOv8. The improved model has the most significant improvement in the small target (person) accuracy compared to the YOLOv5s model, which is 21.2%, and the performance related to all the rest of the categories is better than YOLOv5s. The overall detection accuracy is improved by 1.7%.

While YOLOv7 [37,38] is recognized for its improved detection of small targets in standard image datasets, this advantage is less pronounced in panoramic datasets, particularly when comparing models of equal size. This distinction is reflected in Table 2, where YOLOv7’s performance in detecting the ‘person’ category is notably less effective than expected. It is crucial to highlight that such comparisons are context dependent, and the efficacy of a model can vary significantly with different dataset characteristics. Consequently, we have focused on refining the YOLOv8s model to suit the intricacies of panoramic image analysis better. The enhancements have resulted in a model that is not only more lightweight, but also demonstrates increased accuracy in detecting small targets within panoramic scenes, surpassing the performance of the original YOLOv8s model.

### 5.3. Panoramic Copy–Paste Data-Enhanced Ablation Experiments

For cases with insufficient samples of small targets, we introduced the copy–paste method for panoramic images to expand the labels of small targets. Comparison experiments were conducted under the same conditions.

**Figure 13:**
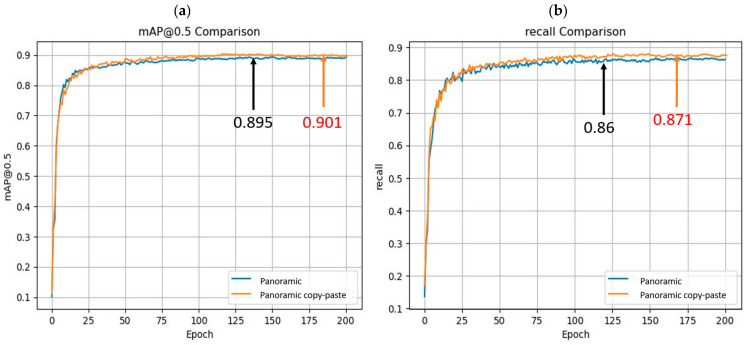
(**a**) Shows the mAP@0.5 comparison between the improved model before and after copy–paste data enhancement, while (**b**) details the corresponding recall comparison. These metrics effectively demonstrate the enhancements in model performance post-data augmentation.

After we made the panoramic dataset, we prepared some small target object pools according to their distance from the center of the panoramic image, scaled them, and randomly rotated them; we randomly chose the objects from the object pools and randomly copied the small targets in the images in the training set, and experimented on the improved model uniformly, as shown in Figure 13. After the data was subject to the copy–paste method, the model mAP@0.5 went up by 0.6%, the recall went up by 1.1%, and the detection precision was improved.

Table 3 shows that the precision of detecting small targets in the panorama image after the improved model and copy–paste data enhancement is still lower than 60%. The small target dataset are not easy to collect, and it takes time to make the dataset. To further improve the accuracy of the small target model detection, we unfold the panoramic image in the same dataset and then bisect and splice it; we achieve the unfolded bisect and spliced image through the mapping relationship between the panoramic image and the unfolded image. The resulting dataset has a better detection effect compared to the unprocessed one, and we have conducted experiments in regard to this. The experimental results are shown in Table 3. The panoramic copy–paste expand is the dataset resulting from the panoramic unfolded bisection stitching.

**Figure 14:**
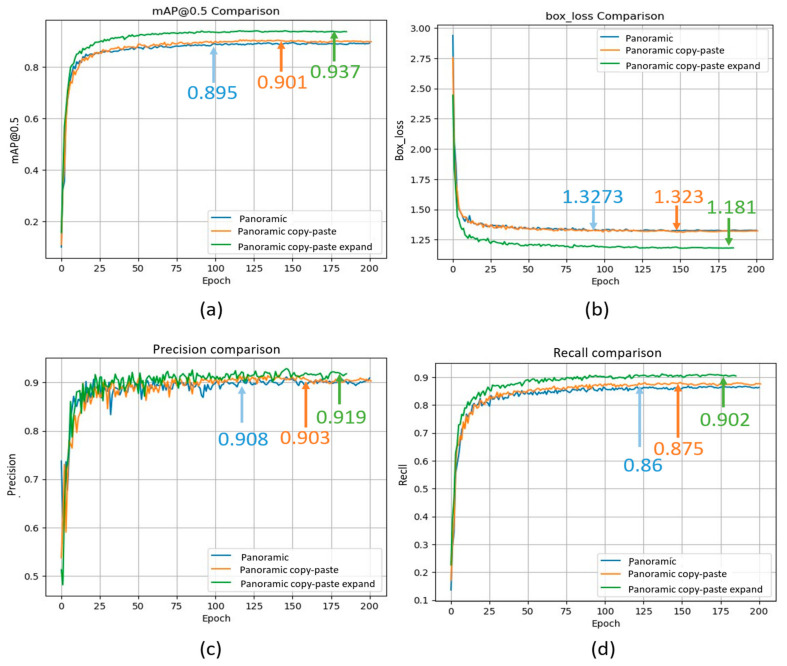
(**a**–**d**) Shows the comparison between the panoramic image dataset and the panoramic processed dataset detecting various evaluation metrics, respectively.

Based on the data in Table 3 and Figure 14, comparing the final results, it can be found that the mAP@0.5 of the processed dataset compared to the panoramic copy–paste expand dataset rises by 4.2% for all target mAP@0.5, 21.3% for the mAP@0.5 of the small targets, and 27.3% for the recall of the small targets. The box_loss is also reduced by 0.0916.

### 5.4. Visualization and Verification

To verify the visual effect of the algorithms in this paper using large-field panoramic images, we collected some panoramic images mounted at a height of 20 m overlooking the ground scene for testing.

**Figure 15:**
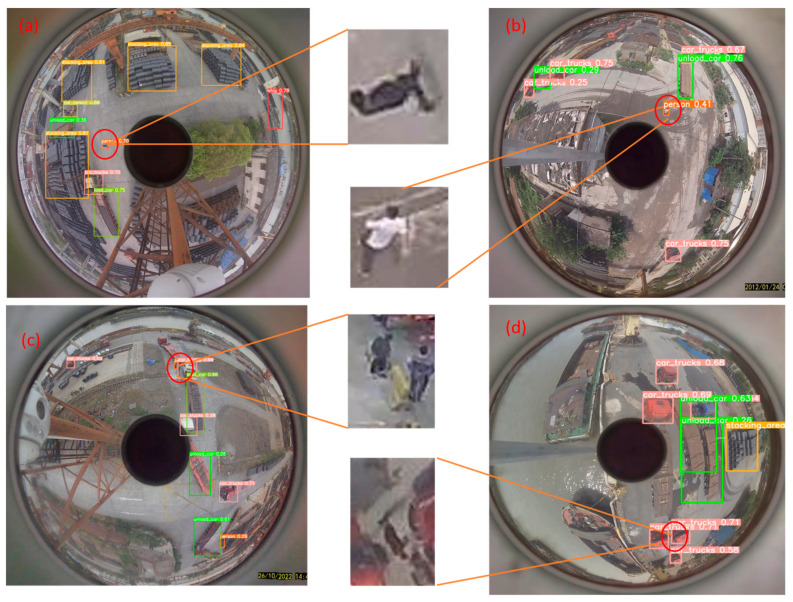
(**a**–**d**) Shows the detection results for the improved model using panoramic images of different scenes.

**Figure 16:**
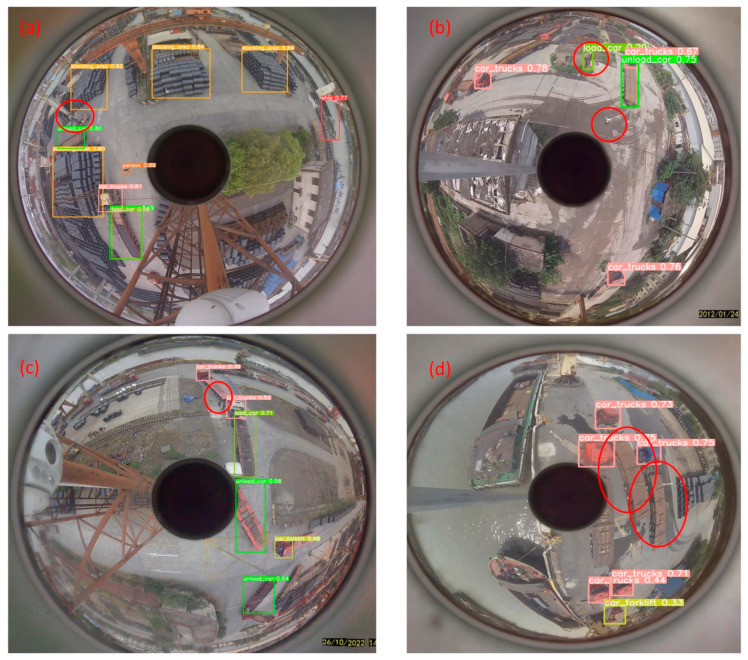
(**a**–**d**) Shows the detection results for the YOLOv8s model using panoramic images of different scenes.

Figure 15 and Figure 16 present our tests on panoramic images across various scenes. Figure 15 illustrates the detections using the improved model in four panoramas labeled a, b, c, and d, where eight types of objects are detected. Notably, the small target people in scenes a and b are successfully detected. However, in Figure 16, which displays two small targets in scene c, only one is detected, and the single small target in scene d is missed. Figure 16 also reveals the detection results using the unimproved YOLOv8s model. The red circles highlight objects that are either missed or incorrectly detected, demonstrating that all small target individuals in scenes b, c, and d remain undetected by the unimproved model.

**Figure 17:**
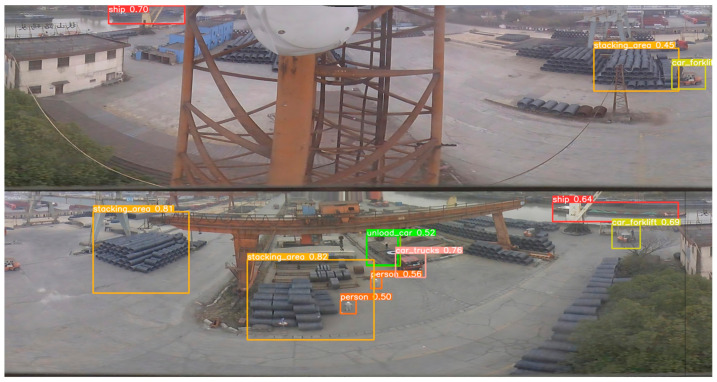
Detection results for the improved model using a preprocessed panoramic image (cloudy).

**Figure 18:**
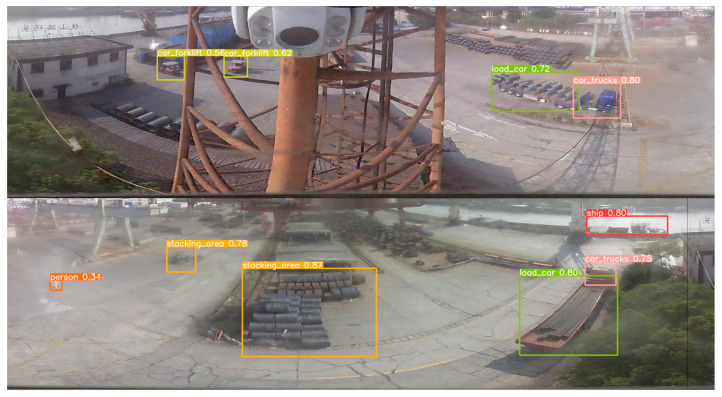
Detection results for the improved model using a preprocessed panoramic image (sunny).

**Figure 19:**
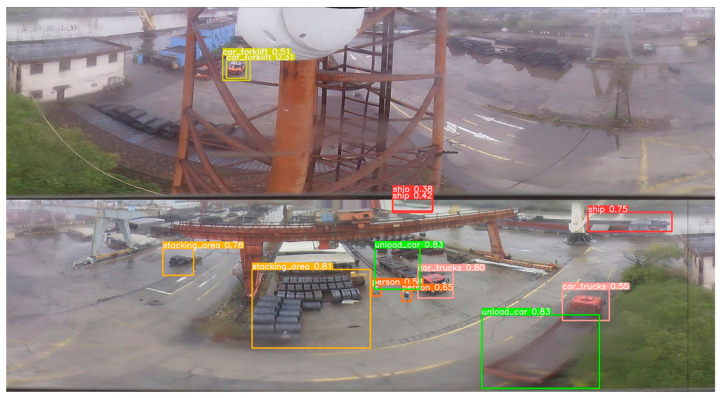
Detection results for the improved model using a preprocessed panoramic image (rainy).

Figure 17, Figure 18 and Figure 19 present a side-by-side analysis of the same scene captured under different weather conditions, allowing for an assessment of how varying lighting and environmental factors impact detection efficacy. Figure 17 depicts the scene on a cloudy day, demonstrating the robustness of the copy–paste data enhancement method in conditions with diffuse lighting. Figure 18 showcases the scene in sunny conditions, where the stark shadows and bright light typically pose challenges for detection algorithms. However, our method continues to identify each individual accurately. Lastly, Figure 19 captures the scene amidst rainfall, where the reduced visibility and reflective surfaces from wet conditions are present, illustrating that our preprocessing technique significantly enhances the accuracy of small target detection across all weather scenarios. The comparative results show that processed panoramic images, adjusted for each specific weather condition, provide more reliable detection outcomes than those achieved by direct detection using the original panoramic images.

## 6. Conclusions

In this paper, for the study of small target detection tasks in wide-area panoramic images, an algorithm YOLOv8s_small_fasterNext_EMA is proposed for small target detection in panoramic images, based on the YOLOv8 network.

In order to resolve the insufficient ability of YOLOv8s in regard to small target detection, a small target detection layer is added to the network to improve the network’s ability to perceive small targets. The EMA mechanism is incorporated into the YOLOv8s backbone network for feature enhancement, suppressing ineffective information in the input features and focusing more on the features of small targets, and the depth of the network is deepened in the network structure, incorporating the attention mechanism and the small-in-small target layer. The parameters are increased and the floating-point arithmetic increases; for this reason, we changed the eighth layer in the YOLOv8s network to the FasterNext module and replaced part of the traditional convolutional CONV with PCONV at the same time, without affecting the detection accuracy, to improve the efficiency of the detection, compared to the YOLOv8s network which is more lightweight. The algorithm in this paper has apparent advantages in regard to wide-area panoramic datasets, and the value of mAP@0.5 is significantly improved compared with the standard model of YOLOv8s and YOLOv7, and the YOLOv5s model with the same network width and depth. The ablation studies on the improved model design reveal that the components of this design significantly enhance detection accuracy, reduce the model’s size, and increase the speed of detection. The visualization experiments show that the wide-area panoramic image of various scenarios resulted in good detection for small targets. However, there is still room for improvement in the presence of occlusion concerning the situation panoramic image detection effect.

Furthermore, this paper introduces a copy–paste data enhancement method for panoramic images, addressing the scarcity of small target sample labels. This method improves the detection of small targets, enhancing both recall and precision. Instead of directly detecting targets in unfolded panoramas, we crop and splice them, resulting in better detection compared to the original panoramic images.

## Figures and Tables

**Table 1:** Improved network modeling for ablation experiments.

Method	mAP@0.5	Parameters	FLOPs (G)	Small Object Category (Person)	Size of Model (MB)
YOLOv8s	87.2%	11.12 M	28.5	36.2%	22.5
YOLOv8s_fasterNext	87.0%	10.09 M	26.8	30.5%	20.4
YOLOv8s_EMA	87.6%	11.14 M	29.3	33.8%	22.5
YOLOv8s_small	89.5%	10.85 M	37.8	53.9%	22.1
YOLOv8s_small_fasterNext	89.9%	9.80 M	36.2	55.2%	20.0
YOLOv8s_small_EMA	90.0%	10.86 M	38.7	57.9%	22.1
YOLOv8s_small_fasterNext_EMA	90.1%	9.82 M	37.7	56.2%	20.1

**Table 2:** Comparison of the performance of other YOLO models and YOLOv8s_ours using the panorama dataset, where a person is the smallest target (tested on RTX3080).

Method	Object Category								mAP@0.5
	Ship	Truck	Person	Stacking	Forklift	Loaders	Un_cart	Car	
YOLOv8s	90.0	92.9	31.7	98.5	94.6	95.4	89.6	98.7	86.4%
YOLOv7	89.8	94.2	30.5	98.5	96.3.	95.9	90.9	99.4	87.0%
YOLOv5s	89.4	91.6	48.4	98.2	93.7	92.6	89.5	98.3	87.8%
YOLOv8_ours	89.5	94.3	52.9	98.7	94.1	95.8	91.4	98.5	89.5%

**Table 3:** Comparison between target detection results for our dataset before and after using data processing.

Dataset	Small Target Train Set	Sample Small Target Test Set	ALL mAP@0.5	Person mAP@0.5	Person Recall
Panoramic	842	866	89.5%	52.9%	38.2%
Panoramic copy–paste	1558	866	90.1%	56.2%	48.5%
Panoramic copy–paste expand	1558	866	93.7%	74.2%	65.5%

## Data Availability

The data presented in this study are available on request from the corresponding author.
